# COVID-19-related delays of botulinum toxin injections have a negative impact on the quality of life of patients with dystonia and spasticity: a single-center ambulatory care study

**DOI:** 10.1007/s00702-021-02430-z

**Published:** 2021-10-23

**Authors:** Yvonne Teuschl, Christian Bancher, Michael Brainin, Alexandra Dachenhausen, Karl Matz, Michaela M. Pinter

**Affiliations:** 1 Department for Clinical Neuroscience and Preventive Medicine, Danube University Krems – University for Continuing Education, Dr. Karl-Dorrek Str. 30, 3500 Krems, Austria; 2 Department of Neurology, Landesklinikum Horn-Allentsteig, Horn, Austria; 3 Department of Neurology, Landesklinikum Baden-Mödling, Mödling, Austria

**Keywords:** Botulinum toxin therapy, Spasticity, Dystonia, Quality of life, COVID-19, Patient rights, Neurorehabilitation

## Abstract

**Background:**

Botulinum toxin A (BoNT-A) is considered a safe and effective treatment for spasticity and dystonia. Individual interinjection intervals are critical for the maintenance of the effect. In Austria, BoNT outpatient clinics were shutdown from November to December 2020 during COVID-19 control measures, leading to rescheduling of BoNT-A injections. This survey aimed at investigating the influence of injection delays on symptoms, physical functioning, and quality of life (QoL) of the affected patients.

**Methods:**

Between April and July 2021, 32 outpatients (21 females, mean age: 63.4 ± 12.1 years) treated ≥ 12 months at the BoNT outpatient clinic Horn-Allentsteig (Austria) and experienced ≥ 2 week injection delays, completed a structured face-to-face questionnaire.

**Results:**

Indications were dystonia (34%), spasticity (63%), and hyperhidrosis (3%). Injections were delayed by 10 weeks (median, range: 2–15). Muscle cramps increased in 95% of patients with spasticity, muscle twitches in 91% of those with dystonia, and pain in 9% and 60% for dystonia and spasticity, respectively. Overall, 75% reported functional worsening, and deterioration in QoL by 62.6% ± 16.8 (mean ± SD). The impact on QoL correlated with the subjective global improvement induced by BoNT-A (Rs: 0.625; *p* < 0.001). For 75%, long-term assurance of BoNT-A therapy was very important, and 81% felt their patient rights not respected.

**Conclusions:**

COVID-19-related delays in BoNT-A injections illustrate the importance of this therapy for symptom relief, functional outcome, and QoL in patients suffering from involuntary muscle hyperactivity. BoNT-A therapy is essential and has to be guaranteed even in circumstances such as the COVID-19 pandemic.

## Introduction

Botulinum toxin (BoNT-A) is used as standard in medical practice for the treatment of spasticity and dystonia as well as for autonomic syndromes (Dressler et al. 2021; Simpson et al. 2016). BoNT-A is a neurotoxic protein produced by the bacterium Clostridium botulinum, which is injected in the affected muscles and causes muscle relaxation, improvement of symptoms, and can thereby facilitate rehabilitation. It is considered safe and effective for the treatment of dystonia and spasticity; however, there is not sufficient evidence to conclude whether BoNT-A can improve the underlying motor dysfunction (Simpson et al. 2016). The relief of symptoms starts approximately 10 days after the injection, with peak effects at 4–6 weeks, and last between 2 and 4 months (Jacinto et al. 2020; Esquenazi et al. 2020). However, longer interval injections may occur in early post-stroke spasticity (Rosales et al. 2018). The maintenance of individual interinjection intervals is critical for functional status and quality of life (QoL) of the affected patients.

In Austria, as in other countries, COVID-19 pandemic countermeasures included shutdowns of all medical activites in hospitals other than for acute life-threatening conditions to protect patients and healthcare providers from the exposure to the disease and to allow shifting staff and facilities to COVID-19 wards and intensive care units. The botulinum toxin outpatient clinic of the hospital Horn-Allentsteig in Austria was closed in November 2020 for 8 weeks, and injections planned for this period had to be rescheduled. After the end of the lockdown, a survey was performed to investigate the influence of injection delays on symptoms and QoL of the affected patients.

## Methods

The botulinum toxin outpatient clinic of the Landesklinikum Horn-Allentsteig treats approximately 700 patients with dystonia, limb spasticity and other indications per year. Due to the COVID-19-related lockdown in November–December 2020, appointments of 50 patients had to be canceled; for 17 of these patients, BoNT-A treatment was delivered during a visit in a neurological practice; however, 33 patients had to be rescheduled until the end of the lockdown. After reopening, all outpatients treated previously for more than 12 months with BoNT-A and experiencing more than 2 weeks of injection delay were invited to complete a structured face-to-face questionnaire during the following visits. Exclusion criteria were serious adverse events such as accidents or severe injuries since the last BoNT-A injection, aphasia making communication impossible, and cognitive impairment (Mini Mental State Examination < 20 points). Informed consent was obtained by the treating physician (MP). The structured questionnaire created by Dressler and Adib Saberi (2020) was used. It includes six questions about changes in spastic or dystonic symptoms, the impact of the delays of injection intervals on QoL, as well as about patients’ perception of the shutdown.

BoNT-A treatment was performed with Incobutulinumtoxin-A (Xeomin®), Onabotulinumtoxin-A (Botox®) or Abobotulinumtoxin-A (Dysport®). For comparison, equivalence mouse units (MU-E) were used, by assuming 1 MU-E = 1 MU Xeomin® = 1 MU Botox® = 1/3 MU Dysport®.

This study has been approved by the local ethics committee of the Danube University Krems (NUMBER EK GZ 42/2018-2021, March, 24, 2021).

### Statistical analysis

Metric data were described by means and standard deviation, median and quartiles as appropriate, and nominal data by relative frequency. To test for the influence of different demographic, disease-related or treatment-related variables on QoL, Mann–Whitney *U* test, or Spearman Rang correlations were used for nominal and metric variables, respectively.

## Results

Between April and July 2021, 32 patients (21 females, 11 males, mean age: 63.4 ± 12.1) were recruited for this survey. All eligible patients agreed to participate. One patient was not invited due to time constraints. The indications for BoNT-A treatment were dystonia in 11 (34.4%), limb spasticity in 20 patients (62.5%), and hyperhidrosis in one (3.1%) patient. Dystonia included blepharospasm (*n* = 6; 54.5%), hemifacial spasm (*n* = 4; 36.4%), and spasmodic torticollis (*n* = 1, 9.1%). Spasticity was hemiparetic in 13 (65%), paraparetic in 3 (15%), and tetraparetic in 4 (20%) patients; the underlying disorders in these patients were ischemic stroke (*n* = 11), hemorrhagic stroke (*n* = 2), multiple sclerosis (*n* = 4), hereditary spastic paralysis (*n* = 2), and unknown (*n* = 1). Overall, patients were treated with BoNT-A in median for 4 years (interquartile range [IQR] 2–8), and before the shutdown, the majority (91%) had interinjection intervals of 12 weeks (median 12, range 8–14 weeks). Most patients were treated with Incobotulinumtoxin-A (78.1%), 15.6% with Onabotulinumtoxin-A and 6.3% with Abobotulinumtoxin-A; the total dose per patient was in median 400 MU-E (IQR: 48–600), but differed according to the affected muscles and thus between patients with dystonia and spasticity (Table 1).

**Table 1:** Impact of COVID-19-related measures on BoNT-A treatment and patients’ quality of life, according to dystonia and spasticity

	Dystonia (*n* = 11)	Spasticity (*n* = 20)	All (*n* = 32)^1^
Female	8 (72.7%)	12 (60.0%)	21 (65.6%)
Age (years, mean ± SD)	66.9 ± 13.4	63.1 ± 9.4	63.4 ± 12.1
Treatment duration (years)	4 (3–18)	3.5 (2–7)	4 (2–8)
Total dose (MU-E)	28 (20–60)	500 (400–650)	400 (48–600)
Clinical Global Improvement (%)	80 (70–100)	80 (50–85)	80 (60–95)
Delay of injection (weeks)	10 (4–11)	11.5 (9.5–12.5)	10 (7.5–12)
Impact on QoL^2^ (mean ± SD)	63.1 ± 16.7	61.7 ± 17.6	62.6 ± 16.8
Subjective decrease of function	9 (90%)	14 (74%)	24 (80%)
Increase in any symptoms	11 (100%)	19 (95%)	31 (97%)
Increase in cramps	1 (9%) ^3^	19 (95%)	
Increase in pain	1 (9%)	12 (60%)	
Increase in twitches	10 (91%)		
Importance of BoNT therapy			
More important than before	11 (100%)	14 (70%)	25 (78%)
Similar	0 (0%)	6 (30%)	7 (22%)
Less important than before	0 (0%)	0 (0%)	0 (0%)
Importance of assurance of therapy			
Very important	10 (91%)	13 (65%)	24 (75%)
Important	1 (9%)	5 (25%)	6 (19%)
Less important	0 (0%)	2 (10%)	2 (6%)
COVID-19 lockdown was			
Adequate	4 (36%)	3 (15%)	7 (22%)
Inadequate	7 (64%)	17 (85%)	25 (78%)
Patient rights were			
Respected	4 (36%)	2 (10%)	6 (19%)
Not respected	7 (64%)	18 (90%)	26 (81%)

*BoNT-A*  botulinum toxin A, *MU-E* equivalent of mouse units, *QoL* quality of life, *SD* standard deviation

Values are numbers (percentages) or median (interquartile range) if not state otherwise

^1^Including the patient with hyperhidrosis

^2^Measured on a visual analogue scale ranging from 0 to 100 with 100 representing the largest impact

^3^Patient with spasmodic torticollis

The injections were delayed by 10 weeks in median (IQR 7.5–12.5) with a maximum of 15 weeks. Overall, 97% of patients reported a worsening of symptoms and 80% reported that this had an impact on their functional status. Muscle cramps increased in 95% of patients with spasticity, muscle contractions in 91% of those with dystonia, and pain increased in 9 and 60% of patients with dystonia and spasticity, respectively. Patient felt that the prolonged interinjection delay decreased their QoL in mean by 62.6 ± 16.8 SD (Table 1).

Patients who rated their subjective improvement induced by BoNT-A larger perceived also the impact of injection delays on their QoL stronger (Rs = 0.664; *p* < 0.001). The impact on QoL was not influenced by sex, age, indication (dystonia or spasticity), treatment duration, total dose, or the delay of the injection (all *p* > 0.3).

**Fig. 1:**
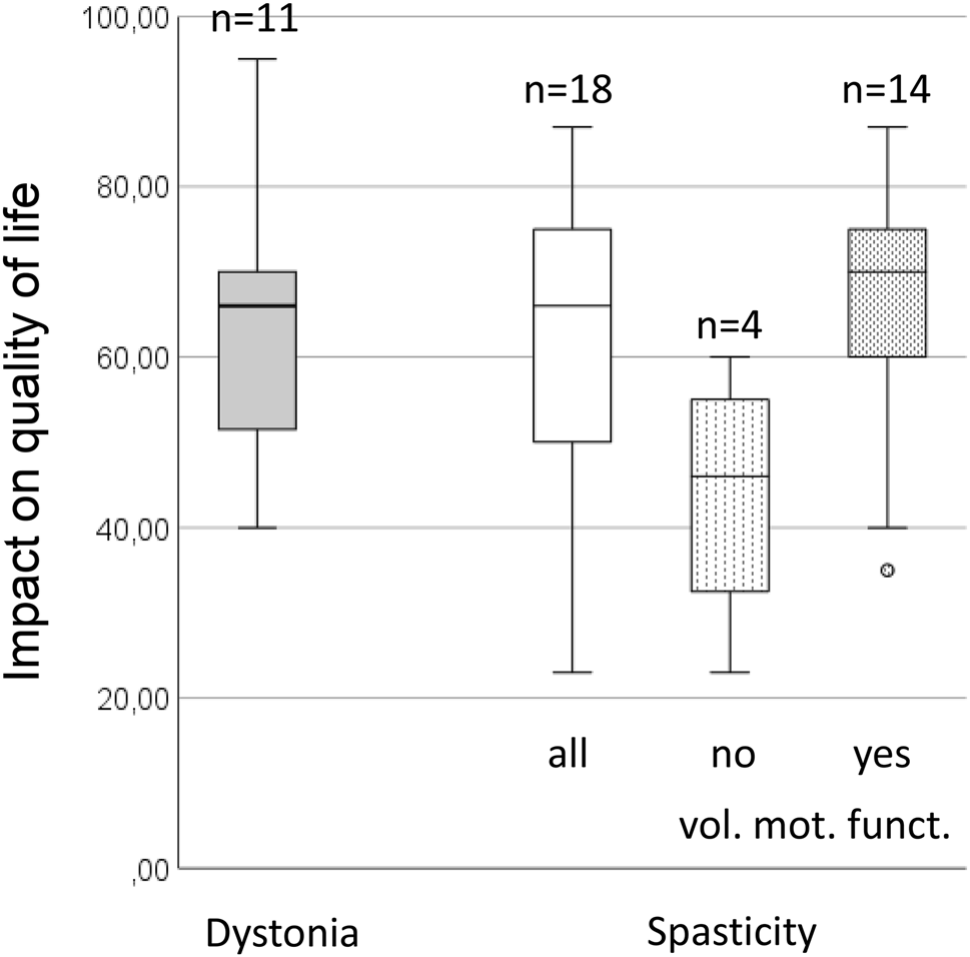
Impact of delays in botulinum toxin injections on quality of life of patients with dystonia, spasticity, and in the subgroups of patients with spasticity with or without voluntary motor function (vol. mot. funct)

In patients suffering from spasticity, the effect on QoL was greater in those with voluntary motor function (*n* = 14, mean 66.8 ± 14.9 SD) compared to those without (*n* = 4, 43.8 ± 15.7; Fig. 1).

After the lockdown, BoNT-A therapy has become more important to 78% of patients. For 75% of patients, long-term assurance of BoNT-A therapy was very important, 78% perceived the COVID-19-related lockdown as inadequate and 81% felt their patient rights not respected (Table 1).

## Discussion

This survey shows how strongly the closure of a botulinum toxin outpatient clinic in Austria and thereby prolonged interinjection intervals affected patients’ symptom reoccurrence and their QoL. Case reports and surveys from Italy, the USA, Germany, and the Philippines have already shown the negative influence of COVID-19-related delays of BoNT-A injections on patients suffering from dystonia, spasticity, or migraine (Dressler and Adib Saberi 2020; Ali 2020; Ranza et al. 2020; Erro et al. 2021; Santamato et al. 2021; Tarantino et al. 2021; Samadzadeh et al. 2021; Pajo et al. 2021). This is underlined by 75–91% of patients perceiving the security of long-term BoNT-A therapy as very important and 76–98% which felt their patient rights not respected by COVID-19 counter measures (this study, Dressler and Adib Saberi 2020; Tarantino et al. 2021).

In our study, the injection delays were in median 10 weeks, i.e., the interinjection intervals were 22 weeks. In a study investigating the perception of BoNT-A therapy in patients with spasticity of different etiology, symptoms re-occurred in mean 12–13 weeks after the injection, with symptoms being mild to moderate at the beginning and more severe on day before the reinjection (Jacinto et al. 2020). Similarly, in a study on Abobotulinumtoxin-A, more than 60% of adult patients with limb spasticity had required reinjections within 12 weeks and all before 24 weeks; only a few patients with cervical dystonia did not require reinjection within 24 weeks (Esquenazi et al. 2020). However, our study included only one patient with cervical dystonia.

An Italian study reported worsening of symptoms in 72%, a negative impact on QoL in 71%, and a loss of independence in 53% of patients after COVID-19-related discontinuation of their usual BoNT-A treatment for spasticity (Santamato et al. 2020). In a German cohort, 44 of 48 (92%) patients reported a worsening of symptoms after delays in reinjection. This is comparable to the 97% found in our study (Samadzadeh et al. 2021). The authors calculated that on average, 1 day of delay caused 1% of worsening compared to the previous visit, with a mean delay of 23 days corresponding to a mean worsening of 26%. In accordance with this, the three times longer delay in our study resulted in a reduction of QoL by 63%. Similarly, Erro et al. (2021) found a mean impact of 5 on 10 points scale after a delay of 10.6 weeks, and Dressler and Adib Saberi (2020) reported a reduction in QoL of 40% after 6.6 weeks. In Italian patients with cerebral palsy, QoL was reduced by 68% after a 9 month delay in BoNT-A therapy (Tarantino et al. 2021).

In our study, patients reported that the reoccurrence of symptoms had a strong impact on participation, for example due to functional blindness or to worsening of walking abilities. The impact of the interinjection delay on QoL was strongest for patients which subjectively profited most from the BoNT-A treatment. QoL was less affected in patients with spastic paresis with no remaining motor function, possibly because benefits of therapy had less influence on their activities of daily life.

Limitations of our study are the low number of participants, the absence of a control group, and that functional status was not assessed objectively. Other factors related to the COVID-19 countermeasures such as cessation of physiotherapy or psychosocial stress could have contributed to the lower QoL. Nevertheless, the waning of BoNT-A effects on symptoms and participation have already been reported before the COVID-19 pandemic (Jacinto et al. 2020; Esquenazi et al. 2020).

Our results emphasize the importance of ensuring the individual BoNT-A interinjection intervals for these patients. This has to be guaranteed even in circumstances such as the COVID-19 pandemic and can be achieved with security measures minimizing the risk of infection (Pajo et al. 2021; Baricich et al. 2020).

## Data Availability

Data are available on request from the authors.
